# Evaluation of C-Reactive Protein/Albumin Ratio in Diabetic Retinopathy Patients

**DOI:** 10.3390/diagnostics15172178

**Published:** 2025-08-28

**Authors:** Şaban Kılıç, Emre Aydın, Çiğdem Deniz Genç

**Affiliations:** Department of Ophthalmology, Samsun Education and Research Hospital, Kısla, Barıs Blv. No:199, 55090 Samsun, Turkey; emreaydin0052@gmail.com (E.A.); cigdemdeniz85@hotmail.com (Ç.D.G.)

**Keywords:** diabetic retinopathy, CRP/albumin ratio, inflammation biomarkers, disease severity, glycemic control

## Abstract

**Purpose:** This study aimed to investigate the relationship between the C-reactive protein/albumin (CRP/albumin) ratio and disease severity in diabetic retinopathy (DR) patients and to evaluate the potential of CRP/albumin as a clinical biomarker for inflammation and DR progression. **Methods:** This single-center, prospective, cross-sectional study included 158 DR patients and 150 healthy controls. Clinical, ophthalmologic, and laboratory evaluations were performed, including best-corrected visual acuity, optical coherence tomography, and measurements of inflammatory and glycemic markers. CRP/albumin ratios were calculated, and their relationships with DR severity were assessed. Receiver operating characteristic (ROC) analysis was performed to evaluate the predictive performance of CRP/albumin. **Results:** Before treatment, CRP/Alb ratios and other inflammatory markers, including NLR, MLR, PLR, and SII, were significantly elevated in DR patients compared to controls. Following treatment, CRP/albumin ratios decreased markedly from 0.14 ± 0.1 to 0.04 ± 0.04 (*p* < 0.001), aligning with improvements in retinal thickness (OCT: 269.2 ± 17.5 µm) and HbA1c levels (6.9 ± 1.2%). CRP levels decreased from 5.8 ± 3.2 mg/L to 1.5 ± 1.4 mg/L, while NLR dropped from 2.5 ± 1.1 to 1.29 ± 0.8 (*p* < 0.001). Significant reductions were also observed in PLR (169.6 ± 62.2 to 128.2 ± 54.3) and SII (743.0 ± 427 to 230.8 ± 187). ROC analysis revealed an AUC of 0.963 for CRP/albumin, indicating high predictive accuracy for DR severity with 95% sensitivity and 90% specificity at a cutoff of 0.052. **Conclusions:** The CRP/albumin ratio is a significant biomarker for inflammation and disease progression in DR. The observed reductions in inflammatory markers post-treatment highlight the importance of inflammation control in DR management.

## 1. Introduction

Diabetes is a chronic metabolic disease with an increasing global prevalence, primarily characterized by uncontrolled hyperglycemia [1]. In the long term, vascular damage caused by hyperglycemia can lead to permanent dysfunction in various organs, including the heart, kidneys, and eyes. However, due to the complex nature of the disease process, it is not always possible to prevent organ damage caused by diabetes [2]. Therefore, the regular monitoring of clinical and biochemical markers plays a crucial role in diabetes management. While controlling blood glucose levels remains the primary monitoring parameter, the importance of new indicators for the early detection of inflammation and vascular changes that emerge as the disease progresses is steadily increasing [3].

Diabetic retinopathy, one of the common microvascular complications of diabetes, develops as a result of structural and functional impairments in the retinal vasculature [4]. The long-term effects of hyperglycemia weaken the endothelial integrity of retinal blood vessels, leading to damage to the blood–retina barrier, abnormal leakage, and ischemia. In advanced stages, proliferative vascular formations and macular edema may occur [5]. This process impairs visual function and may cause permanent blindness. Therefore, the early diagnosis of diabetic retinopathy, prevention of its progression, and development of effective treatment approaches are of great importance for public health.

The role of chronic inflammation in the pathogenesis of diabetic retinopathy has been drawing increasing attention [6]. C-reactive protein (CRP), recognized as a systemic marker of inflammation, is an acute-phase reactant produced in the liver, with its levels rising in response to various tissue-damaging factors [7]. On the other hand, albumin levels provide insights into the body’s nutritional and protein synthesis status, often showing a decline in chronic diseases and various inflammatory conditions. The combined evaluation of CRP and albumin levels offers a more detailed reflection of the degree of inflammation and metabolic stress [8,9]. The CRP/albumin ratio may reflect DR severity and serve as a useful biomarker.

Although the relationship between C-reactive protein (CRP) and diabetic retinopathy (DR) has been investigated in several studies, the results remain inconsistent and certain aspects are still unclear [10,11,12]. Some studies found no significant difference in serum CRP levels between patients with and without DR, whereas others have reported both positive and even inverse associations. In addition, while biomarkers such as the albumin-to-globulin ratio have recently been suggested as useful indicators for DR, the clinical value and mechanisms of composite inflammatory markers (such as the CRP/albumin ratio) in relation to DR severity and response to treatment remain largely unexplored [13]. Furthermore, there is a paucity of prospective studies evaluating changes in systemic inflammation markers before and after anti-VEGF therapy in DR patients. Our study aims to address these gaps by systematically investigating both the association of CRP/albumin ratio with DR severity and the dynamics of inflammatory biomarkers in response to treatment.

## 2. Material and Methods

### 2.1. Study Design and Ethical Approval

The study was conducted in accordance with the Declaration of Helsinki and approved by the Non-Interventional Clinical Research Ethics Committee of Samsun University (approval number REF. No. GOKAEK, approval date 11 April 2024). The study was carried out in accordance with the principles of the Declaration of Helsinki, and informed consent forms were obtained from all participants. The primary aim of the study was to evaluate C-reactive protein (CRP) and albumin levels, as well as their ratio, in patients diagnosed with diabetic retinopathy (DR), thereby elucidating the relationship between these critical parameters reflecting inflammation and nutritional status in the pathogenesis of DR.

### 2.2. Patient and Control Group Selection

The study group consisted of 158 patients diagnosed with diabetic retinopathy (DR). These patients had a diagnosis of either Type 1 or Type 2 diabetes, with a minimum disease duration of 6 months, were over the age of 18, and exhibited various stages of DR without any prior consultation with an ophthalmologist or receiving treatment specific to the retinopathy, thereby ensuring “naive” participants. Demographic characteristics (age, gender), waist circumference, and body mass index (BMI) measurements were recorded, alongside data regarding diabetes type (Type 1 or Type 2) and duration (in months). Patients with chronic liver, kidney, rheumatologic, or autoimmune diseases, active infections, malignancies, regular steroid use, or systemic conditions that could affect laboratory parameters were excluded from the study. Additionally, alcohol and tobacco use were considered exclusion criteria. The control group comprised 150 healthy individuals without a history of diabetes or any chronic systemic diseases, selected with similar age and gender distributions. Ophthalmologic examinations revealed no retinopathy or other ocular pathologies, and their oral glucose tolerance test (OGTT) results were within normal limits. Throughout the study, these patients received intravitreal injections of 0.05 mL bevacizumab (1.25 mg) once a month for three months.

### 2.3. Ophthalmologic Evaluation

All participants underwent a comprehensive ophthalmologic examination. Best-corrected visual acuity (BCVA) was determined using the Snellen chart, anterior segment structures were evaluated via biomicroscopy, and intraocular pressure (IOP) was measured using a Goldmann applanation tonometer. Subsequently, a biomicroscopic fundus examination was performed with a 90-diopter lens to identify diabetes-related lesions, such as microaneurysms, hemorrhages, and exudates, in the retinal vessels and macular region. Optical coherence tomography (OCT) and fundus fluorescein angiography (FFA) were also conducted. These methods allowed for measurements of macular thickness, identification of vascular leakage or ischemic areas, and detection of proliferative formations as signs of diabetic retinopathy (DR). The staging of DR within the study was performed using the International Clinical Diabetic Retinopathy (ICDR) classification, a system that simplifies the Early Treatment Diabetic Retinopathy Study (ETDRS) classification into five main stages to grade the severity of DR and evaluate the presence and extent of macular edema in a similar manner.

### 2.4. Laboratory Analyses

Venous blood samples were collected from all participants in the morning after 8–12 h of fasting. In addition to a complete blood count, glycemic parameters (fasting blood glucose, and HbA1c) and lipid profiles (LDL, HDL, total cholesterol, and triglycerides) were evaluated. CRP and albumin levels, considered key biomarkers reflecting inflammation and nutritional status, were specifically analyzed in the diabetic retinopathy group. CRP levels were measured using high-sensitivity immunoturbidimetric methods, while albumin concentrations were determined through spectrophotometric procedures (BCG or BCP methods). The CRP (mg/L or mg/dL) and albumin (g/dL) values were used to calculate the CRP/albumin ratio, and the relationship of this ratio with the severity of diabetic retinopathy and macular edema was investigated. The dataset was reviewed for additional conditions that might affect the disease (e.g., chronic infections, active inflammation, immune system disorders), and individuals influenced by such factors were excluded from the study.

### 2.5. Statistical Analysis

All statistical analyses were performed using SPSS 27 software (IBM Corp., Armonk, NY, USA). The normality of continuous variables was assessed using the Kolmogorov–Smirnov test. Continuous variables were presented as mean ± standard deviation (mean ± S.D.), while categorical variables were expressed as counts (n) and percentages (%). For continuous variables with parametric distributions, repeated measures over time were analyzed using repeated measures ANOVA (one-way repeated measures analysis of variance). For non-parametric distributions, the changes over time were evaluated using the Friedman test. For comparisons between two groups, the independent samples t-test was used for parametric variables, while the Mann–Whitney U test was applied for non-parametric variables. For multiple group comparisons, one-way ANOVA was used for parametric variables, and the Kruskal–Wallis test was applied for non-parametric variables. Post hoc analyses were conducted to further investigate significant differences. Relationships between categorical variables were evaluated using the Chi-square test, and Fisher’s exact test was applied when necessary. Additionally, the predictive power of biochemical and hematological parameters in the study was assessed using receiver operating characteristic (ROC) analysis. The ROC analysis determined the cut-off values of specific parameters based on sensitivity and specificity, and the area under the curve (AUC) values were calculated. The AUC values were used to evaluate the diagnostic performance of the parameters for diabetic retinopathy. A *p*-value of <0.05 was considered statistically significant in all analyses.

## 3. Results

The participants comprised 46.4% females (n = 143) and 53.6% males (n = 165). Right eye involvement was 37.7% (n = 116), left eye 45.1% (n = 139), and both eyes 17.2% (n = 53). Non-smokers made up 83.4% (n = 257) and smokers 16.6% (n = 51). Non-alcohol users were 95.1% (n = 293), while 4.9% (n = 15) consumed alcohol. Diabetic retinopathy stages were distributed as 13.3% (n = 21) in stage 1, 53.8% (n = 85) in stage 2, 28.5% (n = 45) in stage 3, and 4.4% (n = 7) in stage 4. Regarding treatment, 66.5% (n = 105) were in the treatment group and 33.5% (n = 53) in the other group.

Table 1 presents the demographic characteristics of the study participants. The mean age of all participants was calculated as 65.4 ± 8.4 years. The mean body mass index (BMI) was 25.16 ± 4.5 kg/m^2^, and the average duration of diabetes was determined to be 159.1 ± 77.0 months (Table 1).

The mean age was 65.9 ± 8.1 years in the control group and 64.9 ± 8.7 years in the diabetes mellitus group, with no statistically significant difference between the groups (*p* = 0.298). In terms of gender distribution, 40.7% of the control group and 51.9% of the diabetes mellitus group were female, while 59.3% and 48.1% were male, respectively (*p* = 0.052). The mean body mass index (BMI) was significantly lower in the control group (23.0 ± 2.8 kg/m^2^) compared to the diabetes mellitus group (27.2 ± 4.7 kg/m^2^, *p* < 0.001) (Table 2).

In the control group the neutrophil count was 2.3 ± 0.42, whereas it was measured as 4.24 ± 1.38 before treatment. Following treatment it decreased to 2.81 ± 1.2 in the 1st month and 1.69 ± 0.9 in the 2nd month. Lymphocyte and monocyte levels also decreased similarly after treatment. Fasting blood glucose, which was 85.6 ± 8.6 mg/dL in the control group, was measured as 171.8 ± 59.8 mg/dL before treatment and decreased to 131.4 ± 45.8 mg/dL in the 2nd month post-treatment. LDL, total cholesterol, and triglyceride levels showed a gradual reduction after treatment. HbA1c levels decreased from 8.0 ± 1.4% before treatment to 6.9 ± 1.2% in the 2nd month post-treatment. CRP levels, which were 2.31 ± 0.13 mg/L in the control group, dropped from 5.8 ± 3.2 mg/L before treatment to 1.5 ± 1.4 mg/L in the 2nd month post-treatment. Albumin levels, initially 49.9 ± 2.9 g/dL in the control group, were measured as 40.5 ± 3.0 g/dL before treatment and remained stable at 39.9 ± 3.1 g/dL after treatment. OCT thickness decreased from 333.1 ± 43.9 µm before treatment to 269.2 ± 17.5 µm in the 2nd month post-treatment. All changes in these parameters were statistically significant (*p* < 0.001) (Table 3).

In the control group, the CRP/albumin ratio was 0.046 ± 0.01, whereas it was 0.14 ± 0.1 before treatment (*p* < 0.001). Similarly, the neutrophil-to-lymphocyte ratio (NLR) was 0.86 ± 0.21 in the control group and 2.5 ± 1.1 before treatment. The monocyte-to-lymphocyte ratio (MLR) was 0.13 ± 0.04 in the control group and 0.31 ± 0.1 before treatment, while the platelet-to-lymphocyte ratio (PLR) was 71.9 ± 19.8 in the control group and 169.6 ± 62.2 before treatment. The Systemic Immune-Inflammation Index (SII) was 164.9 ± 55.0 in the control group and 743.0 ± 427 before treatment. All changes were statistically significant (*p* < 0.001) (Table 4).

The CRP/albumin ratio was 0.046 ± 0.01 in the control group, increased to 0.14 ± 0.1 before treatment, and decreased to 0.06 ± 0.06 in the 1st month and 0.04 ± 0.04 in the 2nd month post-treatment (*p* < 0.001). The neutrophil-to-lymphocyte ratio (NLR) was 0.86 ± 0.21 in the control group, rose to 2.5 ± 1.1 before treatment, and decreased to 1.82 ± 1.0 in the 1st month and 1.29 ± 0.8 in the 2nd month. The monocyte-to-lymphocyte ratio (MLR) was 0.13 ± 0.04 in the control group, measured as 0.31 ± 0.1 before treatment, and decreased to 0.3 ± 0.1 in the 1st month and 0.27 ± 0.1 in the 2nd month. The platelet-to-lymphocyte ratio (PLR) was 71.9 ± 19.8 in the control group, increased to 169.6 ± 62.2 before treatment, and dropped to 150.4 ± 58.4 in the 1st month and 128.2 ± 54.3 in the 2nd month. The Systemic Immune-Inflammation Index (SII) was 164.9 ± 55.0 in the control group, rose to 743.0 ± 427 before treatment, and decreased to 441.3 ± 295 in the 1st month and 230.8 ± 187 in the 2nd month. All inflammatory parameters showed statistically significant reductions post-treatment (*p* < 0.001) (Table 5).

The correlation coefficient of the CRP/albumin ratio with HbA1c was calculated as 0.462 and with OCT as 0.431 (*p* < 0.001). The neutrophil-to-lymphocyte ratio (NLR) showed a positive correlation with HbA1c (0.560) and OCT (0.543) (*p* < 0.001). Similarly, the monocyte-to-lymphocyte ratio (MLR) was correlated with HbA1c (0.449) and OCT (0.465) (*p* < 0.001). The platelet-to-lymphocyte ratio (PLR) showed correlations of 0.475 with HbA1c and 0.524 with OCT (*p* < 0.001). For the Systemic Immune-Inflammation Index (SII) significant correlations were observed with HbA1c (0.539) and OCT (0.610) (*p* < 0.001). These results demonstrate significant relationships between inflammatory parameters and both HbA1c and OCT (Table 6).

**Figure 1:**
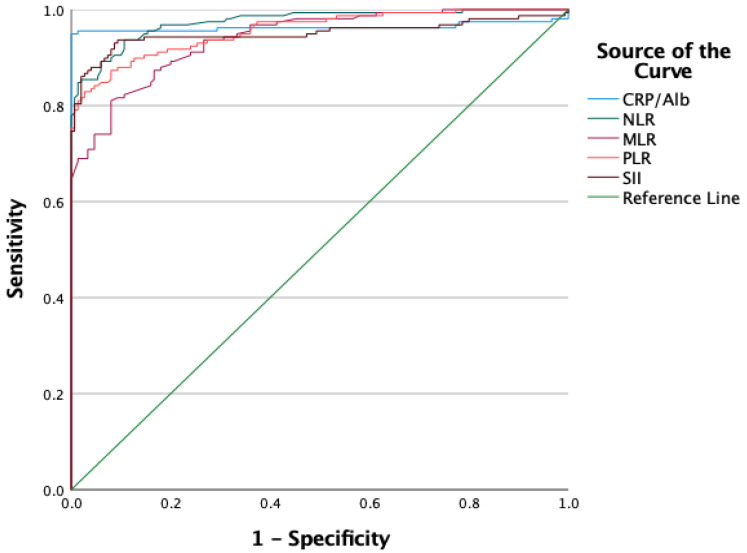
ROC curves of inflammatory parameters for predicting diabetic retinopathy.

Figure 1 illustrates the ROC curves assessing the diagnostic value of CRP/albumin, NLR, MLR, PLR, and SII for predicting the presence of diabetic retinopathy, regardless of its stage. The curves depict the relationship between sensitivity and specificity for each parameter, with the area under the curve (AUC) values demonstrating the diagnostic accuracy of the parameters. The graph highlights that inflammatory parameters are strong predictors for the diagnosis of diabetic retinopathy (Figure 1).

For the CRP/albumin ratio the AUC (area under the curve) value was 0.963, with a cut-off value of 0.052 determined at 95% sensitivity and 90% specificity (*p* < 0.001, 95% CI: 0.935–0.991). The AUC value for NLR was 0.974, with a cut-off value of 1.122 at 97% sensitivity and 89% specificity (*p* < 0.001, 95% CI: 0.959–0.989). For MLR the AUC value was 0.940, with a cut-off value of 0.184 at 85% sensitivity and 84% specificity (*p* < 0.001, 95% CI: 0.916–0.964). The PLR showed an AUC value of 0.956, with a cut-off value of 94.8 at 90% sensitivity and 87% specificity (*p* < 0.001, 95% CI: 0.935–0.977). The AUC value for SII was 0.950, with a cut-off value of 255.6 determined at 95% sensitivity and 91% specificity (*p* < 0.001, 95% CI: 0.922–0.979) (Table 7).

There was a statistically significant increase in CRP/albumin ratio, neutrophil-to-lymphocyte ratio (NLR), monocyte-to-lymphocyte ratio (MLR), platelet-to-lymphocyte ratio (PLR), and Systemic Immune-Inflammation Index (SII) with advancing DR stage (*p* < 0.001 for all parameters) (Table 8).

## 4. Discussion

This study demonstrated that the CRP/albumin ratio is a significant inflammatory marker in diabetic retinopathy and is associated with disease progression. Elevated levels of CRP/Alb, NLR, MLR, PLR, and SII were observed before treatment, which significantly decreased post-treatment, likely due to inflammation control and improved glycemic regulation. This decline supports the role of inflammation in DR and the potential of CRP/albumin as a biomarker. Improvements in inflammatory markers aligned with reductions in OCT-measured retinal thickness and HbA1c levels, emphasizing the importance of inflammation control in disease management.

A study reported by Xiu-Fen Yang et al. highlighted an inverse relationship between increasing CRP levels and diabetic retinopathy progression [11]. However, our findings align with other studies that demonstrate the impact of inflammation on diabetic retinopathy progression and the significant differences in the regulation of inflammatory mediators between diabetic retinopathy patients and those without retinopathy [11,14,15]. Hyperglycemia-driven inflammation may accelerate DR progression. Numerous studies have shown that hyperglycemia induces the production of inflammatory cytokines in various cell types, including endothelial cells [16]. Additionally, Mota et al. (2015) reported increased CRP and TNF-alpha levels in patients with diabetic retinopathy [17]. In our study, significant differences in inflammatory mediators were observed between the PDR (proliferative diabetic retinopathy) and NPDR (non-proliferative diabetic retinopathy) groups. A study by Gustavsson et al. demonstrated a significant correlation between inflammatory mediators and the degree of diabetic retinopathy [18]. It has been proposed that hyperglycemia leads to the activation of TNF-alpha, which is critical in the development and progression of diabetic retinopathy [19,20].

Nalini et al. reported elevated CRP, TNF-alpha, and VEGF levels in DR patients, particularly in the PDR group, supporting the inflammatory nature of disease progression [21]. Our findings align with this, as we observed a reduction in CRP from 5.8 ± 3.2 to 1.5 ± 1.4 mg/L and improvements in HbA1c (6.9 ± 1.2%) and OCT (269.2 ± 17.5 µm) following treatment. Similarly, Dascalu et al. demonstrated significantly higher NLR values in PDR patients and proposed its use as a biomarker for DR progression [22]. Although PLR showed an upward trend, it was not statistically significant. Our results support these findings, with post-treatment declines in CRP/albumin, NLR, PLR, and other inflammatory indices. Both studies emphasize the importance of standardized inflammation-based markers like CRP/albumin, NLR, and PLR in DR monitoring and management. In our study, individuals with additional conditions that could affect CRP levels—such as chronic inflammatory diseases, active infections, autoimmune diseases, and malignancies—were excluded. Therefore, the measured CRP levels are presumed to be largely associated with diabetic retinopathy and its related inflammation. However, whether CRP is an entirely specific biomarker for DR can be determined more definitively through large-scale studies in which additional systemic inflammatory conditions are excluded.

Various studies have attempted to establish cutoff values for NLR and PLR to distinguish between patients with and without DR or to classify patients based on DR severity. These cutoff values were calculated using various statistical methods such as ROC curve analysis, which determines the optimal threshold maximizing sensitivity and specificity. However, the proposed cutoff values have varied significantly, reflecting differences in patient populations, study designs, and statistical methods [23,24]. Furthermore, the relationship between NLR, PLR, and DR is not linear, and some studies suggest that changes in NLR and PLR may be more informative when analyzed as continuous variables rather than categorical variables based on arbitrary thresholds. For instance, higher NLR or PLR values have been associated with increased DR risk or more severe DR. However, the exact risk may depend on other factors such as glycemic control, diabetes duration, and the presence of other microvascular or macrovascular complications [25,26]. In our study, the ROC analysis for CRP/albumin and other inflammatory markers demonstrated high sensitivity and specificity in predicting the presence and severity of DR. For example, the CRP/albumin ratio had an AUC of 0.963, with a threshold value of 0.052 achieving 95% sensitivity and 90% specificity. These findings align with the results of Dascalu et al., further highlighting the potential utility of inflammatory markers as effective biomarkers in the clinical management of DR. However, validating the data obtained through ROC analysis in larger and more diverse populations is critical to standardizing these parameters.

There is emerging evidence that anti-VEGF therapy not only reduces ocular angiogenesis and edema but may also lead to decreases in systemic inflammatory markers. For example, in patients with neovascular age-related macular degeneration significant reductions have been observed in inflammatory parameters, such as C-reactive protein (CRP), neutrophil-to-lymphocyte ratio (NLR), and CRP/albumin ratio (CAR), after a three-month loading dose of aflibercept injections compared to pre-treatment levels [27]. Similarly, in patients with macular edema secondary to central retinal vein occlusion, marked decreases in serum levels of systemic inflammatory indicators such as NLR, platelet-to-lymphocyte ratio (PLR), and Systemic Immune-Inflammation Index (SII) have been reported following intravitreal ranibizumab treatment [28]. These findings suggest that, beyond their local effects, anti-VEGF agents may also modulate inflammatory processes and thus reduce systemic inflammation in conditions such as diabetic retinopathy. However, larger-scale studies are needed to fully elucidate the impact of anti-VEGF therapy on systemic inflammation.

This study has some limitations. First, the patient population was drawn from a single center, which may limit the generalizability of the findings. Although our study employed a prospective design, the sample size was relatively small, potentially restricting the statistical power of the results. Additionally, we did not perform pharmacokinetic or bioavailability analyses of intravitreal bevacizumab, such as measuring serum or intraocular drug concentrations, which limited our ability to directly assess the relationship between systemic drug exposure and changes in inflammatory parameters. Future studies including pharmacokinetic and bioavailability data may further elucidate these associations. One of the strengths of our study is that it is among the first in the literature to systematically examine how inflammation changes with treatment in diabetic retinopathy (DR) patients. In this respect, our research provides novel and important insights into the role of inflammation in DR pathogenesis and the improvements observed during treatment. Furthermore, analyses based on easily measurable biomarkers such as the CRP/albumin ratio offer a new perspective for monitoring treatment efficacy and evaluating disease progression. Our use of a prospective design ensured that data were collected in a timely and controlled manner, which enhances the reliability and statistical strength of the findings. Additionally, the application of advanced statistical methods such as ROC analysis strongly supports the potential of inflammatory markers to predict the presence and severity of DR. The significant reductions observed in inflammatory markers post-treatment underscore the importance of targeting inflammation in DR management and make a valuable contribution to the existing literature.

## Figures and Tables

**Table 1:** Demographic characteristics of participants.

	Mean ± S.D.
Age (years)	65.4 ± 8.4
BMI (kg/m^2^)	25.16 ± 4.5
Duration of Diabetes (months)	159.1 ± 77.0

All values are presented as mean ± S.D. Abbreviations: BMI, body mass index.

**Table 2:** Baseline demographic characteristics according to group.

		Control	Pre-Treatment	*p* Value
Age (years)		65.9 ± 8.1	64.9 ± 8.7	0.298
Gender	Female	61 (40.7)	82 (51.9)	0.052
	Male	89 (59.3)	76 (48.1)	
BMI (kg/m^2^)		23.0 ± 2.8	27.2 ± 4.7	**<0.001**

All parameters are presented as mean ± standard deviation (S.D.) or n (%), as appropriate. Abbreviations: BMI, body mass index. Statistically significant *p*-values are shown in bold.

**Table 3:** Hematological and biochemical parameters before and after treatment.

	Control	Pre-Treatment	Post-Treatment 1. Month	Post-Treatment 2. Month	
	Mean ± S.D.	Mean ± S.D.	Mean ± S.D.	Mean ± S.D.	*p* Value
Neutrophil (10^9^/L)	2.3 ± 0.42	4.24 ± 1.38	2.81 ± 1.2	1.69 ± 0.9	**<0.001**
Lymphocyte (10^9^/L)	2.76 ± 0.46	1.87 ± 0.5	1.68 ± 0.5	1.43 ± 0.4	**<0.001**
Monocyte (10^9^/L)	0.36 ± 0.1	0.53 ± 0.14	0.45 ± 0.12	0.36 ± 0.1	**<0.001**
Platelet (10^9^/L)	193.3 ± 44.4	291.7 ± 57.7	232.5 ± 52.0	168.1 ± 44.2	**<0.001**
Glucose (mg/dL)	85.6 ± 8.6	171.8 ± 59.8	154.6 ± 53.8	131.4 ± 45.8	**<0.001**
LDL (mg/dL)	106.1 ± 13.9	127.9 ± 26.8	115.6 ± 24.3	98.3 ± 20.6	**<0.001**
HDL (mg/dL)	49.4 ± 6.0	47.7 ± 8.1	45.3 ± 7.8	45.3 ± 7.7	**<0.001**
T. Chol. (mg/dL)	177.3 ± 14.8	203.7 ± 37.0	183.8 ± 33.0	156.2 ± 28.0	**<0.001**
TG (mg/dL)	96.8 ± 28.9	140.3 ± 55.7	119.5 ± 47.4	95.6 ± 37.9	**<0.001**
HbA1c (%)	5.1 ± 0.3	8.0 ± 1.4	7.61 ± 1.4	6.9 ± 1.2	**<0.001**
CRP (mg/L)	2.31 ± 0.13	5.8 ± 3.2	2.2 ± 2.5	1.5 ± 1.4	**<0.001**
Albumin (g/dL)	49.9 ± 2.9	40.5 ± 3.0	39.9 ± 3.1	39.9 ± 3.1	**<0.001**
OCT (μm)	251.0 ± 12.8	333.1 ± 43.9	326.8 ± 25.0	269.2 ± 17.5	**<0.001**

All parameters are presented as mean ± standard deviation (S.D.). Comparisons were made among the four groups: control, pre-treatment, post-treatment 1st month, and post-treatment 2nd month. The *p*-values indicate the overall statistical significance of differences across all groups for each parameter (using repeated measures ANOVA or the appropriate non-parametric test). Abbreviations: LDL, low-density lipoprotein; HDL, high-density lipoprotein; T. Chol., total cholesterol; TG, triglyceride; HbA1c, hemoglobin A1c; CRP, C-reactive protein; OCT, optical coherence tomography. Statistically significant *p*-values are shown in bold.

**Table 4:** Inflammatory parameters before and after treatment.

	Control	Pre-Treatment	
	Mean ± S.D.	Mean ± S.D.	*p* Value
CRP/Albumin	0.046 ± 0.01	0.14 ± 0.1	**<0.001**
NLR	0.86 ± 0.21	2.5 ± 1.1	**<0.001**
MLR	0.13 ± 0.04	0.31 ± 0.1	**<0.001**
PLR	71.9 ± 19.8	169.6 ± 62.2	**<0.001**
SII	164.9 ± 55.0	743.0 ± 427	**<0.001**

All parameters are presented as mean ± standard deviation (S.D.). Abbreviations: CRP/Albumin, C-reactive protein to albumin ratio; NLR, neutrophil-to-lymphocyte ratio; MLR, monocyte-to-lymphocyte ratio; PLR, platelet-to-lymphocyte ratio; SII, Systemic Immune-Inflammation Index. Statistically significant *p*-values are shown in bold.

**Table 5:** Inflammatory parameters and their progression before and after treatment.

	Control	Pre-Treatment	Post-Treatment 1. Month	Post-Treatment 2. Month	
	Mean ± S.D.	Mean ± S.D.	Mean ± S.D.	Mean ± S.D.	*p* Value
CRP/Albumin	0.046 ± 0.01	0.14 ± 0.1	0.06 ± 0.06	0.04 ± 0.04	**<0.001**
NLR	0.86 ± 0.21	2.5 ± 1.1	1.82 ± 1.0	1.29 ± 0.8	**<0.001**
MLR	0.13 ± 0.04	0.31 ± 0.1	0.3 ± 0.1	0.27 ± 0.1	**<0.001**
PLR	71.9 ± 19.8	169.6 ± 62.2	150.4 ± 58.4	128.2 ± 54.3	**<0.001**
SII	164.9 ± 55.0	743.0 ± 427	441.3 ± 295	230.8 ± 187	**<0.001**

All parameters are presented as mean ± standard deviation (S.D.). Abbreviations: CRP/Albumin, C-reactive protein to albumin ratio; NLR, neutrophil-to-lymphocyte ratio; MLR, monocyte-to-lymphocyte ratio; PLR, platelet-to-lymphocyte ratio; SII, Systemic Immune-Inflammation Index. Statistically significant *p*-values are shown in bold.

**Table 6:** Correlation between inflammatory parameters and HbA1c and OCT.

		HbA1c (%)	OCT (μm)
CRP/Albumin	Pearson Correlation	0.462	0.431
	Sig. (2-tailed)	**<0.001**	**<0.001**
NLR	Pearson Correlation	0.560	0.543
	Sig. (2-tailed)	**<0.001**	**<0.001**
MLR	Pearson Correlation	0.449	0.465
	Sig. (2-tailed)	**<0.001**	**<0.001**
PLR	Pearson Correlation	0.475	0.524
	Sig. (2-tailed)	**<0.001**	**<0.001**
SII	Pearson Correlation	0.539	0.610
	Sig. (2-tailed)	**<0.001**	**<0.001**

Correlation coefficients were calculated using Pearson correlation analysis. Abbreviations: CRP/Albumin, C-reactive protein to albumin ratio; NLR, neutrophil-to-lymphocyte ratio; MLR, monocyte-to-lymphocyte ratio; PLR, platelet-to-lymphocyte ratio; SII, Systemic Immune-Inflammation Index; HbA1c, glycated hemoglobin; OCT, optical coherence tomography. Statistically significant *p*-values are shown in bold.

**Table 7:** ROC analysis results of inflammatory parameters for predicting diabetic retinopathy.

	Area	Sensitivity	Specificity	Cut-Off	*p* Value	Asymptotic 95% CI	
						Lower B.	Upper B.
CRP/Alb.	0.963	%95	%90	0.052	**<0.001**	0.935	0.991
NLR	0.974	%97	%89	1.122	**<0.001**	0.959	0.989
MLR	0.940	%85	%84	0.184	**<0.001**	0.916	0.964
PLR	0.956	%90	%87	94.8	**<0.001**	0.935	0.977
SII	0.950	%95	%91	255.6	**<0.001**	0.922	0.979

Abbreviations: CRP/Alb., C-reactive protein to albumin ratio; NLR, neutrophil-to-lymphocyte ratio; MLR, monocyte-to-lymphocyte ratio; PLR, platelet-to-lymphocyte ratio; SII, systemic immune-inflammation index; CI, confidence interval. Statistically significant *p*-values are shown in bold. AUC: Area under the ROC curve; unitless value (range: 0–1).

**Table 8:** Inflammatory parameters according to diabetic retinopathy stages.

	Stage 1 (n = 21)	Stage 2 (n = 85)	Stage 3 (n = 45)	Stage 4 (n = 7)	
	Mean ± S.D.	Mean ± S.D.	Mean ± S.D.	Mean ± S.D.	*p* Value
CRP/Albumin	0.10 ± 0.04	0.14 ± 0.1	0.17 ± 0.1	0.21 ± 0.02	**<0.001**
NLR	1.48 ± 0.6	2.22 ± 0.8	3.07 ± 1.1	4.57 ± 0.9	**<0.001**
MLR	0.20 ± 0.05	0.28 ± 0.1	0.38 ± 0.1	0.54 ± 0.1	**<0.001**
PLR	125.4 ± 24.0	153.6 ± 40.8	198.9 ± 64.9	308.6 ± 75.5	**<0.001**
SII	365.6 ± 148.2	629.5 ± 227.3	979.4 ± 448.7	1734.0 ± 508.8	**<0.001**

All parameters are presented as mean ± standard deviation (S.D.). Abbreviations: CRP/Albumin, C-reactive protein to albumin ratio; NLR, neutrophil-to-lymphocyte ratio; MLR, monocyte-to-lymphocyte ratio; PLR, platelet-to-lymphocyte ratio; SII, Systemic Immune-Inflammation Index. Statistically significant *p*-values are shown in bold.

## Data Availability

The data presented in this study are not publicly available due to ethical and privacy restrictions. However, anonymized data may be made available from the corresponding author upon reasonable request.
